# Metabolic changes in cancer cells upon suppression of *MYC*

**DOI:** 10.1186/2049-3002-1-7

**Published:** 2013-02-04

**Authors:** Elena Anso, Andrew R Mullen, Dean W Felsher, José M Matés, Ralph J DeBerardinis, Navdeep S Chandel

**Affiliations:** 1 Department of Medicine, Feinberg School of Medicine, Northwestern University, 60611, Chicago, IL, USA; 2 Children’s Medical Center Research Institute, University of Texas Southwestern Medical Center at Dallas, 75390, Dallas, TX, USA; 3 Departments of Medicine and Pathology, Division of Oncology, Stanford University School of Medicine, 94305, Stanford, CA, USA; 4 Department of Molecular Biology and Biochemistry, Faculty of Sciences, University of Málaga, 29071, Málaga, Spain

**Keywords:** *MYC*, Glutaminolysis, Glucose, Glutamine, Oncogene addiction, Mitochondrial ROS, Mitochondrial metabolism

## Abstract

**Background:**

Cancer cells engage in aerobic glycolysis and glutaminolysis to fulfill their biosynthetic and energetic demands in part by activating *MYC*. Previous reports have characterized metabolic changes in proliferating cells upon *MYC* loss or gain of function. However, metabolic differences between *MYC*-dependent cancer cells and their isogenic differentiated counterparts have not been characterized upon *MYC* suppression *in vitro*.

**Results:**

Here we report metabolic changes between *MYC*-dependent mouse osteogenic sarcomas and differentiated osteoid cells induced upon *MYC* suppression. While osteogenic sarcoma cells increased oxygen consumption and spare respiratory capacity upon *MYC* suppression, they displayed minimal changes in glucose and glutamine consumption as well as their respective contribution to the citrate pool. However, glutamine significantly induced oxygen consumption in the presence of *MYC* which was dependent on aminotransferases. Furthermore, inhibition of aminotransferases selectively diminished cell proliferation and survival of osteogenic sarcoma *MYC*-expressing cells. There were minimal changes in ROS levels and cell death sensitivity to reactive oxygen species (ROS)-inducing agents between osteoid cells and osteogenic sarcoma cells. Nevertheless, the mitochondrial-targeted antioxidant Mito-Vitamin E still diminished proliferation of *MYC*-dependent osteogenic sarcoma cells.

**Conclusion:**

These data highlight that aminotransferases and mitochondrial ROS might be attractive targets for cancer therapy in *MYC*-driven tumors.

## Findings

### Background

Tumor cells display increased glucose metabolism to meet the anabolic demands required for cell proliferation [1]. Glycolytic intermediates fuel anabolic pathways that synthesize NADPH, ribose, phospholipids, triacylglycerols, and serine [2]. However, aerobic glycolysis by itself is not able to supply all the metabolites required for cell proliferation. The mitochondrial tricarboxylic acid cycle (TCA cycle) provides additional metabolites that funnel into lipid, amino acid and nucleotide synthesis. TCA cycle-dependent biosynthesis requires constant replenishment of carbons into the TCA via multiple anaplerotic pathways [3]. Pyruvate derived from glucose can provide acetyl-CoA and oxaloacetate to initiate the TCA while glutamine can also replenish the TCA metabolites through the process of glutaminolysis, where glutamine is converted to glutamate by glutaminases (GLSs), which then enters the TCA cycle by conversion into alpha-ketoglutarate by the aminotransferases or glutamate dehydrogenase [4]. Additionally, glutamine serves as an important nitrogen donor for assembly of amino acids, nucleotides and nicotinamide. Collectively, the metabolism of glucose and glutamine can provide nearly all the necessary carbon and nitrogen required for optimal cell proliferation and growth.

In the past decade, there have been multiple mechanisms uncovered that regulate the metabolism of glucose and glutamine for cell proliferation in normal and cancer cells [5,6]. One mechanism, by which both normal and cancer cells meet their metabolic demands for cell proliferation, is through activation of *c-MYC* (herein termed *MYC*) [7]. Normal cells induce *MYC* upon cell surface receptor-dependent signaling to stimulate aerobic glycolysis and glutaminolysis to promote cell proliferation, while cancer cells have deregulated *MYC* allowing proliferation to occur in a cell-autonomous manner [8,9]. For example, *MYC* increases glycolysis in part through the regulation of lactate dehydrogenase A (LDHA) and glutaminolysis by upregulating expression of GLS [10-12]. *MYC* also regulates mitochondrial metabolism through induction of genes such as *TFAM*, which is required for replication and maintenance of mitochondrial DNA and mitochondrial biogenesis [13]. A consequence of increased mitochondrial metabolism is the generation of reactive oxygen species (ROS) that are required to drive tumorigenesis [14,15].

Much of our understanding of *MYC* has come from examining metabolic pathways required for cell proliferation upon *MYC* loss or gain of function [12,13,16]. In the current study we took the opposite approach - whereby metabolic changes were examined when *MYC* was suppressed in osteosarcoma cells highly dependent on *MYC* for their tumorigenic potential [17]. *MYC* suppression in these genetically engineered mouse osteosarcoma cells results in differentiation into osteocytes. This allowed us to compare metabolic differences between differentiated and proliferating cells of the same genetic background. Our results indicate the induction of glutaminolysis as the major metabolic difference observed between *MYC*- dependent osteosarcoma cells and osteocytes. Furthermore, mitochondrial-targeted antioxidants diminished proliferative capacity of osteosarcoma cells without having detrimental effects on osteocytes.

### Methods

#### Cell culture and reagents

*MYC*-dependent osteogenic sarcoma cells were isolated from a transgenic mouse as previously described [17]. These cells in the presence of doxycycline undergo differentiation into osteocytes. Osteosarcoma cells were cultured in high glucose Dulbecco’s modification of Eagle’s medium (DMEM) supplemented with 5% penicillin/streptomycin, 10% fetal bovine serum (FBS) and HEPES buffer. For nutrient deprivation experiments, glucose and glutamine-free DMEM was supplemented with 10% dialyzed serum in the presence of 10 mM glucose and/or 4 mM glutamine. Rotenone, antimycin A, oligomycin, FCCP, aminooxyacetic acid (AOA), N-acetylcysteine (NAC), beta-phenylethyl isothiocyanate (PIETC) and buthionine sulfoximine (BSO) were purchased from Sigma (St. Louis, MO, USA). Antibodies against *MYC* and actin were purchased from Santa Cruz Biotechnology (Santa Cruz, CA, USA). Antibodies against VDAC1, GOT2 and GPT2 were purchased from Abcam (Cambridge, MA, USA). Antibody against GLS1 was prepared in the Matés laboratory.

#### Oxygen consumption rate

Oxygen consumption rate (OCR) was measured using the 24 well Extracellular Flux Analyzer XF24 (Seahorse Bioscience, North Billerica, MA, USA) according to the manufacturer’s protocol. Cells were equilibrated with DMEM lacking bicarbonate and HEPES at 37°C for one hour in an incubator lacking CO_2_. Basal OCR was measured followed by sequential treatments with oligomycin A (5 μM), carbonyl cyanide 4-(trifluoromethoxy)phenylhydrazone (FCCP, 10 μM) and antimycin A (2 μM) + rotenone (2 μM). Measurements were normalized to cell number in each well. A minimum of four wells were utilized per condition in any given experiment. The spare respiratory capacity was calculated as previously described [18].

#### ROS measurement

Mitochondrial ROS production was measured using a redox sensitive GFP probe (roGFP2) targeted to the mitochondrial matrix or cytosolic compartments. Cells were infected with adenovirus containing roGFP2 as previously described [19]. As internal controls, samples were fully reduced with 1 mM dithiothreitol (DTT) and fully oxidized with 1 mM H_2_O_2_. Upon oxidation the roGFP2 gains excitability at 405 nm while losing excitability at 488 nm. Percent oxidized probe was determined with the equation:

(R-R_DTT_)/(R-R_H2O2_)

where R is sample without DTT or H_2_O_2_ added; R_DTT_ fully reduced sample, and R_H2O2_ is fully oxidized.

#### Cell cycle analysis and death

Cells were trypsinized and fixed in ethanol 70% overnight at −20°C. Subsequently, cells were resuspended in a PBS solution containing 50 μg/mL propidium iodide (PI) and 0.1 mg/mL RNase A and incubated 40 minutes at 37°C. Then, the cell pellet was resuspended in PBS and analyzed using a FACS flow-cytometer (Becton Dickinson, Franklin Lakes, NJ USA). Cell death was determined by incubating cells in 0.1 μg/mL PI. Data were analyzed with CellQuest software.

#### Mitochondrial membrane potential

Cells were stained with 100 nM tetramethylrhodamine, ethyl ester (TMRE) for 30 minutes in PBS at 37°C. The cells were trypsinized and washed with PBS. As control, cells were treated with the uncoupling agent FCCP at 50 μM for 10 minutes before staining. Median fluorescence intensity (MFI) values were corrected by FCCP background in each cell type. Data were analyzed in a Beckton Dickinson LSR Fortessa cell analyzer (Franklin Lakes, NJ USA and analyzed with FlowJo (Ashland, OR, USA) software.

#### Metabolic assays

Concentrations of glucose, lactate, and glutamine were determined by incubating cells in DMEM with 10% dialyzed FBS and supplemented with 10 mM D-glucose and 2 mM L-glutamine. After six hours, 0.6 mL aliquots of medium were analyzed using an automated electrochemical analyzer (BioProfile Basic-4 analyzer; NOVA Biomedical, Waltham, MA, USA). Metabolic rates were determined by normalizing absolute changes in metabolite abundances to protein content as previously described [20]. Isotopic labeling was performed in DMEM with 10% dialyzed FBS supplemented with either 10 mM D-[U-^13^C]glucose and 2 mM L-glutamine, or 2 mM L-[U-^13^C]glutamine and 10 mM D-glucose. After six hours, metabolites were extracted with 50% methanol and analyzed using an Agilent 6970 gas chromatograph and an Agilent 5973 (Santa Clara CA, USA) mass selective detector. Analysis of ^13^C enrichment and mass isotopomer distribution was performed as previously described [21].

#### Statistical analysis

*P-*values associated with all pairwise comparisons were based on Student’s *t*-test for independent groups. Error bars were defined using standard error of the mean (SEM).

### Results and discussion

#### Osteogenic sarcoma cells differentiate into osteocytes upon MYC suppression

**Figure 1:**
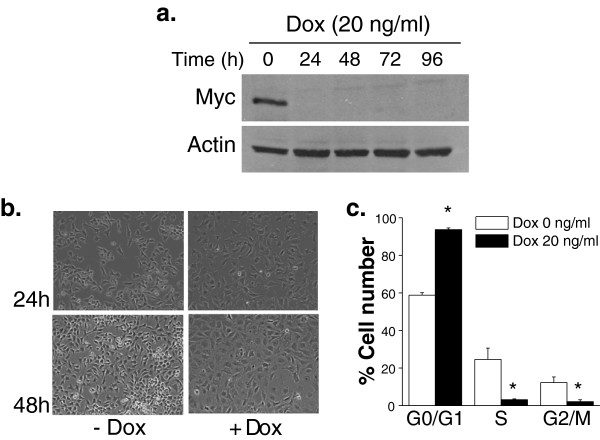
**Osteogenic sarcoma cells differentiate into mature osteocytes under *MYC *suppression. (a)** MYC protein levels in osteogenic sarcoma cells treated with doxycycline (20 ng/mL) at 24, 48, 72 and 96 hours. **(b)** Phase contrast microscopy pictures of osteogenic sarcoma cells after 24 and 48 hours treatment with doxycycline (20 ng/mL). **(c)** Cell cycle analysis was performed by staining the cells with propidium iodide (PI) after 48 hours doxycycline (20ng/ml) treatment. N = 4 ± SEM * *P* < 0.05 compared to 0 ng/ml doxycycline.

Previous reports have utilized inducible systems to examine the effects of *MYC* on metabolism using inducible systems in immortalized proliferating cells [13]. We were interested in using a conditional system examining metabolic differences upon *MYC* suppression in a tumor cell background. This would permit us to characterize, in a unique system, the metabolic differences between cancer cells and their isogenic differentiated counterparts. Previously, an inducible *MYC*-dependent mouse osteogenic sarcoma cell line was characterized, which upon doxycycline treatment, becomes a differentiated osteocyte [17]. We also find that these mouse osteosarcoma cells, upon suppression of their MYC protein levels, display morphological changes and an inhibition of proliferation consistent with a differentiation phenotype (Figure 1).

#### Osteocytes have increased mitochondrial oxygen consumption compared to MYC-dependent osteogenic sarcoma cells

**Figure 2:**
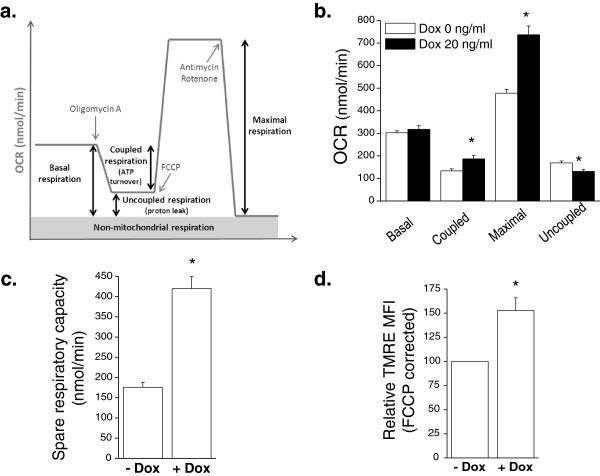
**Bioenergetic profiles of osteocytes and *MYC***-**dependent osteogenic sarcoma cells. (a)** Oxygen consumption rate (OCR) was determined using a Seahorse Bioscience XF24 Flux Analyzer by sequential injection of oligomycin, FCCP and antimycin A/rotenone. **(b)** Basal, coupled, maximal, and uncoupled oxygen consumption rate (OCR) was assessed in osteogenic sarcoma cells in the presence or absence of 20 ng/mL doxycycline for 48 hours. N = 6 ± SEM * *P* < 0.05 compared to 0 ng/ml doxycycline. **(c)** Spare respiratory capacity defined as maximal respiration minus basal respiration. N = 6 ± SEM * *P* < 0.05 compared to 0 ng/ml doxycycline. **(d)** Mitochondrial membrane potential as assessed by TMRE mean fluorescence intensity (MFI), corrected by FCCP. N = 5 ± SEM * *P*<0.05 compared to 0 ng/ml doxycycline.

Previous reports indicate that *MYC* induction can stimulate oxygen consumption [13]. However, these studies compared metabolic differences between proliferating cells with or without *MYC*. We calculated basal mitochondrial respiration by measuring oxygen consumption and subtracting the residual oxygen consumption in the presence of electron transport complex I and III inhibitors rotenone and antimycin, respectively (Figure 2a). Coupled respiration indicates the rate of mitochondrial oxygen consumption utilized to generate ATP by the mitochondrial F1Fo-ATPase (Figure 2a). The basal respiration subtracted from the residual respiration upon the addition of oligomycin, an F1Fo-ATPase inhibitor, allowed us to calculate the rate of coupled respiration. Maximal respiration can be calculated by the addition of FCCP, a potent protonophore which uncouples mitochondrial ATP generation from oxygen consumption (Figure 2a). Osteocytes displayed an increase in coupled and maximal respiration compared to osteogenic sarcoma cells (Figure 2b). The spare respiratory capacity, a measure of how effectively the electron transport chain can respond to energy demand, was also substantially elevated in osteocytes compared to osteogenic sarcoma cells (Figure 2c). Mitochondrial membrane potential is another measure of mitochondrial fitness. Mitochondrial membrane potential allows efficient import and export of proteins to the mitochondrial matrix. Mitochondria that lose their membrane potential undergo mitophagy. We utilized the TMRE fluorescent dye to assess mitochondrial membrane potential and subtracted the fluorescence upon the addition of FCCP, which dissipates the mitochondrial membrane potential. Osteocytes had a substantial increase in mitochondrial membrane potential compared to *MYC*-dependent osteogenic sarcoma cells (Figure 2d). These data indicate that, overall, differentiated osteocytes have better bioenergetic capacity than *MYC*-dependent osteogenic sarcoma cells.

#### Glucose and glutamine stimulate glycolysis and respiration respectively in MYC-dependent osteogenic sarcoma cells

**Figure 3:**
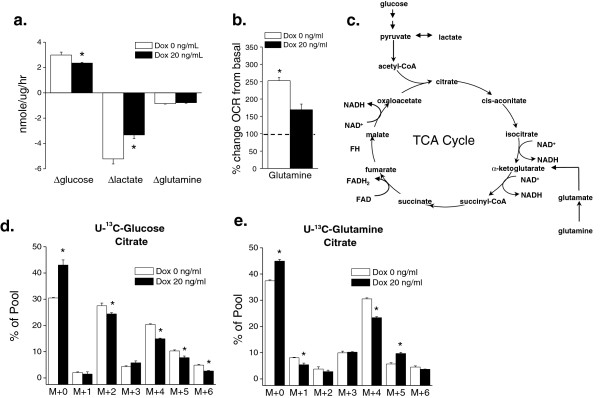
**Glucose and glutamine**-**dependent metabolism in osteocytes and *MYC***- **dependent osteogenic sarcoma cells.** Doxycycline (20 ng/mL) was added to *MYC*- dependent osteogenic sarcoma cells and incubated for 48 hours. **(a)** Glucose and glutamine consumption along with lactate production was measured at six hours. N = 3 ± SEM * *P*<0.05 compared to 0 ng/ml doxycycline. **(b)** Cells were incubated in glutamine-free media for one hour. OCR was determined by injection of glutamine (4 mM). N = 5 ± SEM * *P*<0.05 compared to 20 ng/ml doxycycline. **(c)** Glucose and glutamine carbons feed into the TCA cycle. **(d)** and **(e)** Doxycycline was added to cells (0 or 20 ng/mL) and incubated for 48 hours and then labeled for six hours with either U^13^-Glucose or U^13^-Glutamine and subsequently citrate pools were examined. N = 3 ± SEM * *P*<0.05 compared to 0 ng/ml doxycycline.

*MYC*-dependent osteogenic sarcoma cells displayed a slight elevation in glucose consumption and lactate production compared with osteocytes (Figure 3a). Although there was no change in overall glutamine consumption between osteogenic sarcoma cells and osteocytes (Figure 3a), glutamine substantially stimulated OCR in osteogenic sarcoma cells compared to osteocytes (Figure 3b), suggesting an impairment of entry of glutamine-derived carbon into the TCA cycle when *MYC* expression was curtailed. We incubated cells without glutamine for one hour and subsequently measured basal respiration followed by addition of glutamine. *MYC* increases cell proliferation in osteogenic sarcoma cells, thereby placing a heavy demand for mitochondrial TCA cycle metabolites for macromolecule synthesis. In particular, citrate is exported from the mitochondria and utilized for fatty acid synthesis in *MYC*-dependent cancer cells. Glutamine fuels mitochondria through the process of glutaminolysis, where carbons from glutamine enter the citric acid cycle through conversion to glutamate, and subsequently to alpha-ketoglutarate. The alpha-ketoglutarate undergoes decarboxylation by alpha-ketoglutarate dehydrogenase to eventually regenerate oxaloacetate (OAA) pools. This process generates the reducing equivalents NADH and FADH2, both of which donate electrons to the mitochondrial respiratory chain and consequently drive oxygen consumption (Figure 3c). Oxaloacetate combines with acetyl-CoA to generate citrate, initiating another round of oxidative metabolism to generate precursors for the synthesis of lipids and other macromolecules. Because many nutrients, including glucose and glutamine, can supply acetyl-CoA and/or oxaloacetate, we performed ^13^C tracing experiments to define the specific contribution of glucose and glutamine to these pools. When cells were cultured with [U-^13^C]-glucose and unlabeled glutamine for six hours, the osteocytes displayed a significant increase in the unlabeled citrate fraction (m+0) and a decrease in glucose derived m+2, m+4, and m+6 pools indicating that over this time period, a lower fraction of the citrate pool was supplied by glucose metabolism. To examine the effect of *MYC* suppression on glutamine’s contribution to citrate formation, we cultured both cell lines with [U-^13^C]-glutamine and unlabeled glucose and examined ^13^C enrichment in citrate. Citrate m+4 is formed when glutamine-derived alpha-ketoglutarate is metabolized in the TCA cycle to generate OAA m+4; this can combine with an unlabeled acetyl-CoA to form citrate with four additional mass units. We observed a modest increase in the unlabeled fraction, and a decrease in the amount of glutamine-derived citrate m+4 in the osteocytes (Figure 3e). Interestingly, there was an increase in glutamine-derived citrate m+5 when *MYC* was suppressed. This suggests that osteocytes engage in glutamine-dependent reductive carboxylation, a phenomenon observed in brown fat, cancer cells with defective mitochondria or under hypoxia [21-24]. This is consistent with the higher ability of glutamine to activate respiration in *MYC*-expressing cells (Figure 3b). Glutamine-induced respiration is likely a combined result of reducing equivalents generated during the conversion of glutamine to OAA for anaplerosis, plus the enhanced entry of ^13^C-labeled, glucose-derived acetyl-CoA into the citric acid cycle when *MYC* is expressed (Figure 3d). Osteocytes, which do not engage in proliferation, may use glutamine for other processes in addition to anaplerosis.

#### MYC-dependent osteogenic sarcoma cells are dependent on glucose and glutamine compared to osteocytes

**Figure 4:**
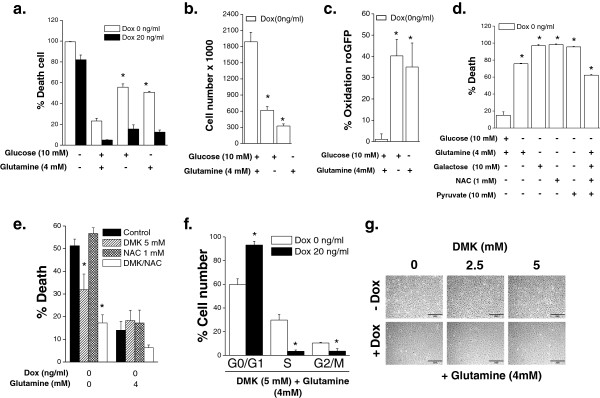
***MYC***-**dependent osteogenic sarcoma cells are dependent on glucose and glutamine for survival.** Doxycycline (20 ng/mL) was added to *MYC*-dependent osteogenic sarcoma cells and incubated for 48 hours. Subsequently, cells were placed in complete media or in media depleted of glucose, glutamine or both. **(a)** Cell death was assessed after 48 hours. N = 3 ± SEM * *P* < 0.05 compared to media containing both glucose and glutamine. **(b)** Cell number was assessed after 24 hours. N = 3 ± SEM * *P* < 0.05 compared to media containing both glucose and glutamine. **(c)** Cytosolic ROS was measured after 24 hours using roGFP2. N = 4 ± SEM * *P* < 0.05 compared to media containing both glucose and glutamine. **(d)** Cell death was assessed in osteogenic sarcoma cells depleted with glutamine and supplemented with galactose (10 mM), pyruvate (5 mM) and/or NAC (1 mM). N = 3 ± SEM * *P* < 0.05 compared to media containing both glucose and glutamine. **(e)** Cell death was assessed in osteogenic sarcoma cells depleted with glutamine and supplemented with DMK (5 mM) and/or NAC (1 mM). N = 4 ± SEM * *P* < 0.05 compared to media containing no glucose and no glutamine. **(f)** Cell cycle analysis at 48 hours in osteogenic sarcoma cells simultaneously treated with DMK and doxycycline. N = 3 ± SEM * *P* < 0.05 compared to 0 ng/ml doxycycline. **(g)** Phase contrast microscopy pictures of osteogenic sarcoma cells treated with DMK (0–5 mM), glutamine (4 mM) and doxycycline.

Previous data indicate that *MYC*-dependent cancer cells are dependent on glucose and glutamine for proliferation and survival [25]. In agreement with these previous studies, we observed that osteogenic sarcoma cells underwent a substantial decrease in cell proliferation and increase in cell death upon glucose or glutamine deprivation (Figure 4a and 4b). By contrast, MYC-deprived osteocytes displayed a slight increase in cell death upon glucose or glutamine deprivation (Figure 4a). To understand the mechanism underlying the induction of cell death upon glucose or glutamine deprivation in *MYC*-dependent osteogenic sarcoma cells, we examined the levels of ROS, which can induce cell death at high levels. One of the primary mechanisms by which cells regulate ROS levels is through detoxification by glutathione. However, NADPH is required to maintain this redox balance by reducing oxidized glutathione. Importantly, glucose and glutamine catabolism support NADPH production through pentose phosphate pathway and glutaminolysis, respectively. Thus, the deprivation of these nutrients could increase ROS levels, contributing to the increase in cell death. Indeed, osteogenic sarcoma cells starved for glucose or glutamine induced a dramatic increase in the oxidation of cytosolic redox-sensitive GFP (roGFP), indicative of an increase in cytosolic ROS levels (Figure 4c). To determine whether the ROS were required for this increase in cell death, we administered the antioxidant NAC upon glucose or glutamine deprivation. However, NAC did not prevent cell death due to glucose deprivation (Figure 4d). Interestingly galactose, which mainly supports cell proliferation by entering into the pentose phosphate pathway and makes cells exclusively rely on oxidative phosphorylation for ATP generation [26], could not substitute glucose (Figure 4d). This is in contrast to oncogenic Kras-driven tumor cells which can rely on galactose [14]. Also, NAC did not diminish cell death upon glutamine deprivation. However, NAC in combination with cell-permeable dimethyl alpha-ketoglutarate (DMK) diminished cell death (Figure 4e). DMK in the presence of glutamine did not prevent cell cycle arrest upon doxycycline administration, suggesting that providing the end product of glutaminolysis is not sufficient to prevent cell cycle arrest that accompanies the transition of *MYC*-dependent osteogenic sarcoma cells into osteocytes (Figure 4f and 4g).

#### MYC-dependent osteogenic sarcoma cells are dependent on aminotransferases

**Figure 5:**
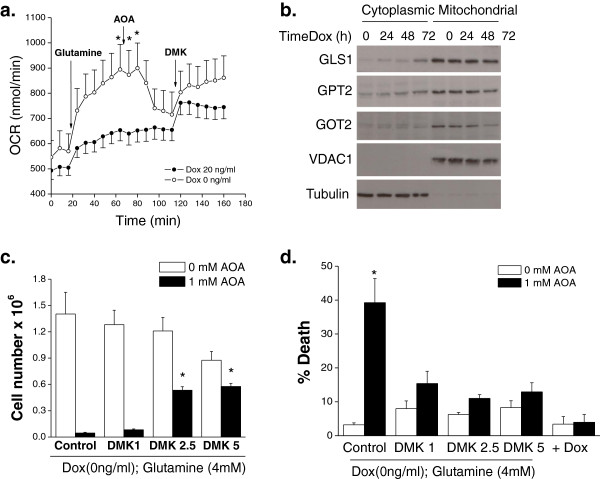
***MYC***-**dependent osteogenic sarcoma cells are dependent on aminotransferases.** Doxycycline (20 ng/mL) was added to *MYC*-dependent osteogenic sarcoma cells and incubated for 48 hours. **(a)** Cells were incubated without glutamine for one hour. OCR was measured and subsequently after the addition of glutamine (4 mM) followed by AOA (1 mM) and DMK (5 mM). N = 7 ± SEM * *P* < 0.05 compared to no glutamine. **(b)** Protein expression of GLS1, GPT2, and GOT2 in mitochondrial and cytoplasmic fractions in cells treated with doxycycline. VDAC1 and tubulin are loading controls for mitochondrial and cytosolic fractions, respectively. **(c)** Cell proliferation was assessed after 48 hours treatment with AOA (1 mM). N = 3 ± SEM * *P* < 0.05 compared to 0 mM DMK with 1 mM AOA. **(d)** Cell death assessed after 48 hours of treatment with AOA (1 mM) in the presence of DMK (0–5 mM). N = 6 (control condition) and N = 3 (for experimental conditions) ± SEM * *P* < 0.05 compared to 0 mM AOA.

In *MYC*-overexpressing cells glutaminase is upregulated, favoring the conversion of glutamine to glutamate. Subsequently, aminotransferases or glutamate dehydrogenase (GDH) convert glutamate into alpha-ketoglutarate to fuel the TCA cycle. To test whether aminotransferases are required for glutamine-dependent increase in OCR in *MYC*-dependent osteogenic sarcoma cells, we utilized AOA, an inhibitor of aminotransferase activity. Glutamine substantially stimulated OCR which was diminished by AOA and rescued by DMK (Figure 5a). Interestingly, GLS1 protein levels in the mitochondrial fractions did not change upon doxycycline addition while the protein levels of the aminotransferase GOT2 in the mitochondrial fractions substantially diminished. GPT2, the other aminotransferase in the mitochondria, slightly diminished upon doxycycline addition (Figure 5b). AOA induced cell death and decreased cell proliferation, which was rescued by DMK in the *MYC*-dependent osteogenic sarcoma cells (Figure 5c and 5d). AOA had minimal effect on OCR and cell death in the differentiated osteocytes (Figure 5a and 5d). Collectively, these results indicate that *MYC*-dependent cancer cells have an increased dependence on aminotransferases when compared to their isogenic differentiated counterparts.

#### MYC-addicted osteogenic sarcoma cells are dependent on mitochondrial ROS for cell proliferation

**Figure 6:**
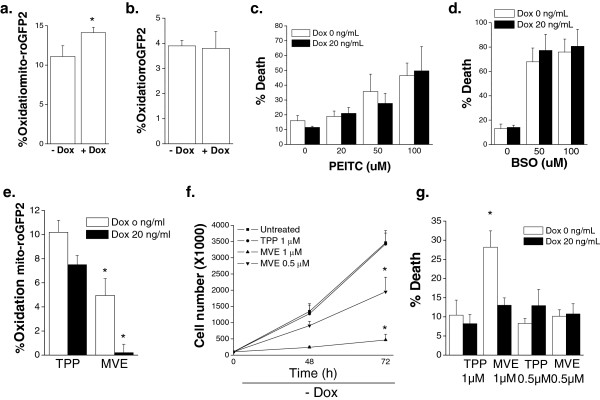
***MYC***-**dependent osteogenic sarcoma cells are dependent on mitochondrial ROS for cell proliferation.** Doxycycline (20 ng/mL) was added to *MYC*-dependent osteogenic sarcoma cells and incubated for 48 hours. **(a)** Mitochondrial ROS was assessed using oxidation of mitochondrial targeted roGFP2. N = 4 ± SEM * *P*<0.05 compared to without doxycycline. **(b)** Cytosolic ROS was assessed using oxidation of cytosolic roGFP2. N = 4 ± SEM. **(c)** Cells were treated with PEITC (0 to 100 μM) for 24 hours and cell death was measured. N = 4 ± SEM. **(d)** Cells were treated with BSO (0 to 100 uM) for 24 hours and cell death was measured. N = 4 ± SEM **(e)** Mitochondrial ROS as assessed by mito-roGFP2 in cells treated with control TPP (0.5 μM) or mitochondrial targeted vitamin E (MVE 0.5 μM). N = 6 ± SEM * *P*<0.05 compared to TPP control. **(f)** Cell proliferation was assessed upon treatment with TPP (1 μM) or MVE (0.5 or 1 μM) for 48 and 72 hours. N = 3 ± SEM * *P*<0.05 compared to 1 mM TPP control. **(g)** Cell death was assessed after 48 hours of treatment with TPP or MVE. N = 4 ± SEM * *P*<0.05 compared to 1 mM TPP control without doxycycline.

Previously, we and others have demonstrated that *MYC* or oncogenic Ras-dependent cancer cells have higher levels of ROS compared to their isogenic immortalized cells [14,27,28]. At low levels ROS can be utilized to activate signaling pathways such as AKT and mitogen-activated protein (MAP) kinases to provide necessary proliferative, growth and survival signals for tumorigenesis. Furthermore, ROS can activate transcription factors such as nuclear factor kappa beta (NF-KB) and hypoxia inducible factors (HIFs) which are important for metabolic adaptation and survival. Antioxidants which decrease ROS levels can prevent *MYC*-dependent tumorigenesis in part by inhibiting HIFs [29]. By contrast, increasing ROS levels through various mechanisms including inhibiting glutathione synthesis, selectively induces cancer cell death since cancer cells have higher basal levels of ROS compared to normal cells. We analyzed cytosolic ROS and mitochondrial ROS levels with a cytosolic-targeted roGFP2 probe and a mitochondrial-targeted roGFP2 (mito-roGFP2) in *MYC*-dependent osteogenic sarcoma cells and osteocytes. This redox-sensitive GFP probe contains two reactive cysteine thiols located on the outer surface. Hydrogen peroxide can oxidize these thiols while DTT can reduce these thiols. roGFP2 emission at 525 nm is assessed via flow cytometry at excitation wavelengths at 405nm and 488 nm. The excitation fluorescence at 405 nm increases while the excitation at 488 nm decreases when the probe is oxidized. The ratio between 405 and 488 allows the GFP probe signal to be independent of the protein expression within cells. Both cell types displayed similar levels of cytosolic roGFP oxidation but the osteocytes displayed a slightly elevated mitochondrial roGFP oxidation (Figure 6a and 6b). This is consistent with higher oxygen consumption and mitochondrial membrane potential in osteocytes (Figure 2). Interestingly, depletion of glutathione by PEITC or BSO did not selectively induce cell death in *MYC*-dependent osteogenic sarcoma cells (Figure 6c and 6d). However, diminishing mitochondrial ROS levels by using a mitochondrial-targeted vitamin E (MVE) in *MYC*-dependent osteogenic sarcoma cells did decrease cell proliferation (Figure 6e and 6f). MVE is targeted to mitochondria by covalently coupling the vitamin E moiety to a triphenylphosphonium cation (TPP), which is used as control [30,31]. TPP is a cation that is rapidly taken up into the mitochondrial matrix due to the negative mitochondrial membrane potential. MVE induced cell death in the osteogenic sarcoma cells compared to the control TPP compound (Figure 6g). MVE significantly diminished ROS levels but did not cause significant cell death in the differentiated osteocytes compared to the control TPP compound (Figure 6e and 6g). These data demonstrate that mitochondrial ROS are critical for proliferation and survival of *MYC* cancer cells.

### Conclusions

*MYC*’s regulation of cellular metabolism in a cell-autonomous manner has been primarily elucidated by using immortalized proliferating cells with inducible expression of *MYC*, mitogenic stimulation of quiescent primary fibroblasts or by utilizing naive T cells isolated from wild-type or *c-MYC* null mice. However, the metabolic differences between isogenic *MYC*-dependent cancer cells and their differentiated counterparts have not been studied. Furthermore, the metabolic transitions that accompany exit from the cell cycle have only rarely been evaluated. Our present results indicate that *MYC*-dependent osteosarcoma cells are dependent on glucose metabolism through glycolysis for survival compared to osteocytes, their differentiated counterparts. Glutamine-induced mitochondrial respiration is necessary for cell proliferation and survival of *MYC*-dependent cancer cells compared to their differentiated counterparts. This is consistent with previous studies where MYC induces glucose and glutamine-dependent metabolism to sustain the anabolic demands due to cell proliferation. Aminotransferases were required for glutamine to sustain mitochondrial metabolism in *MYC*-dependent cancer cells, consistent with previous findings that these enzymes predominate over glutamate dehydrogenase in glucose-consuming, proliferating cancer cells [32]. Interestingly, the inhibition of these enzymes results in cell death of the *MYC*-dependent cancer cells but not their differentiated counterparts. We had previously shown that oncogenic Kras-dependent cells require aminotransferases to sustain mitochondrial metabolism and cell proliferation [14]. Furthermore, inhibition of aminotransferases prevents xenograft tumor growth of MDA-MB-231 breast cancer cells and *MYC*-dependent neuroblastoma cells [33,34]. This raises the possibility that inhibition of aminotransferases might be an effective strategy to inhibit *MYC*-dependent tumorigenesis.

Previous studies have suggested that tumor cells have higher levels of ROS compared to normal cells. ROS, in addition to causing genomic instability, can also increase tumorigenesis by activating signaling pathways that regulate cellular proliferation, angiogenesis, and metastasis [35]. The higher levels of ROS in cancers cells can be exploited as an effective selective strategy to kill tumor cells over normal cells. The administration of agents such as PIETC or BSO, which disable antioxidant defense mechanisms in cells, raises ROS to intolerable levels in cancer cells but not to normal cells which exhibit lower basal ROS levels [27]. However, we did not notice substantial differences in ROS levels between *MYC*- dependent cancer cells and their differentiated counterparts and PIETC or BSO did not have any selective killing of *MYC*-dependent cancer cells. Nevertheless, *MYC*-dependent tumor cells were dependent on mitochondrial ROS for cell proliferation as mitochondrial targeted antioxidant MVE drastically reduced cell proliferation. While previous studies have demonstrated that NAC is effective in reducing *MYC*-dependent tumorigenesis [29], it remains to be determined whether mitochondrial-targeted antioxidants would also prevent tumorigenesis *in vivo*.

## Abbreviations

DMEM: Dulbecco’s modification of Eagle’s medium; FBS: fetal bovine serum; HEPES: (4-(2-hydroxyethyl)-1-piperazineethanesulfonic acid; FCCP: Trifluorocarbonylcyanide phenylhydrazone; AOA: aminooxyacetic acid; NAC: N-acetylcysteine; PIETC: beta-phenylethyl isothiocyanate; BSO: buthionine sulfoximine; VDAC1: voltage dependent anion channel 1; GOT2: glutamic oxaloacetic transaminase 2; GPT2: glutamic pyruvate transaminase 2; ROS: reactive oxygen species; roGFP2: redox sensitive green fluorescent protein 2; mito-roGFP2: mitochondrial targeted roGFP2; OCR: oxygen consumption rate; GFP: green fluorescent protein; DTT: dithiothreitol; TCA: tricarboxylic acid cycle; TMRE: tetramethylrhodamine, ethyl ester; TFAM: mitochondrial transcription factor A; MVE: mitochondrial targeted vitamin E; OAA: oxaloacetate; GLSs: glutaminases

## Competing interests

The authors declare that they have no competing interests.

## Authors’ contributions

EA technically performed the experiments in Figures 1 and 2 and 4, 5, 6. ARM performed the experiments in Figure 3. DWF provided the *MYC*-dependent osteogenic sarcoma cells and carefully edited the manuscript. JMM provided the antibody against GLS1 and carefully edited the manuscript. RJD designed the experiments and edited the paper. NSC and EA designed the experiments and wrote the paper. All authors read and approved the final manuscript.
